# Perceived Concerns and Psychological Distress of Healthcare Workers Facing Three Early Stages of COVID-19 Pandemic

**DOI:** 10.3389/fpsyg.2022.742810

**Published:** 2022-03-10

**Authors:** María Cristina Richaud, Leandro Eidman, Jael Vargas Rubilar, Viviana Lemos, Belén Mesurado, María Carolina Klos, Marisa Rodriguez de Behrends, Rubén N. Muzio

**Affiliations:** ^1^Consejo Nacional de Investigaciones Científicas y Técnicas (CONICET), Buenos Aires, Argentina; ^2^Centro Interdisciplinario de Investigaciones en Ciencias de la Salud y del Comportamiento (CIICSAC), Universidad Adventista del Plata (UAP), Libertador San Martín, Argentina; ^3^Instituto de Ciencias para la Familia (ICF), Universidad Austral (UA), Buenos Aires, Argentina; ^4^Equipo GPS Salud, Buenos Aires, Argentina; ^5^Departamento de Investigación en Ciencias Sociales, Universidad de Ciencias Empresariales y Sociales (UCES), Buenos Aires, Argentina; ^6^Instituto de Filosofía, Universidad Austral (UA), Buenos Aires, Argentina; ^7^Instituto de Biología y Medicina Experimental (IBYME-CONICET), Buenos Aires, Argentina; ^8^Instituto de Investigaciones, Facultad de Psicología, Universidad de Buenos Aires (UBA), Buenos Aires, Argentina

**Keywords:** healthcare workers, COVID-19, comparison of perceived concerns according to quarantine stage, psychological distress, mental wellbeing

## Abstract

**Background:**

This study analyzed the difference in psychological distress of the healthcare workers in three different periods of the coronavirus disease 2019 (COVID-19) pandemic in Argentina. Specifically, from the third week of the mandatory quarantine through the two following weeks.

**Methods:**

Analysis of the responses of 1,458 members of the health personnel was done on a questionnaire on healthcare workers concerns regarding the care of patients with coronavirus, indicators of depression, anxiety, intolerance of uncertainty, and coping.

**Results:**

The psychological indicators that were considered presented differences between the evaluated periods. Perceived concerns about the possibility of infecting loved ones and infecting themselves were greatest in the periods after the onset of the pandemic. In addition, the perception of how the work environment worsened and how lack of sleep interfered with their work was also higher in periods 2 and 3. The same results were found in the indicators of depression, anxiety, and intolerance of uncertainty. Finally, the indicators of high tension and concurrent lack of emotional control, which was greater in the last periods evaluated, were also expressed in the coping strategies (showing emotional lability, only contained by hypercontrol).

**Conclusion:**

The differences found in the psychological indicators between the evaluated periods support the need for early psychological care of health personnel which should be a priority of public health and a fundamental fact to increase its immediate effectiveness in the care of infected patients.

## Introduction

Mental health in disaster situations, like the current pandemic outbreak of coronavirus disease 2019 (COVID-19), has become an important field for the development of scientific knowledge to face the moments in which a large number of people have their lives severely affected by natural catastrophes and man-made catastrophes (Bolton and Tang, 2004; Silove and Steel, 2006). The impact of the disasters differs according to the type, speed, and scale of the catastrophe and to the social, historical, and cultural context in which they take place (Ozer et al., 2003; Porter and Haslam, 2005). However, disasters have some key elements in common. Particularly, the threat they represent to human survival and adaptation. Moreover, despite the cultural differences, individuals and communities manifest some universal patterns of psychosocial response (Green, 1996; Weiss et al., 2003). Therefore, when planning mental health initiatives after a disaster, it is important to optimize the emergent knowledge about these psychological reactions and how these shape the need for adequate mental health services (Silove, 2005).

In the present COVID-19 pandemic, several psychosocial issues with relevant consequences in terms of world mental health (including depression, anxiety, intolerance of uncertainty, and coping, among others) have progressively emerged throughout time while diseases continue spreading (Satici et al., 2020; Weibelzahl et al., 2021).

Depression is an emotional state that is habitually low, accompanied by the loss of the previous ability to enjoy and be interested in daily activities and things the person used to like and be interested in before the depression. It usually comes with irritability, extreme and persistent fatigue, sleep problems, changes in eating habits, difficulty in focusing and making decisions, and feelings of uselessness and blame. From a cognitive point of view, there is a negative way of thinking that is more or less generalized, regarding the self, others, the world, the future, the environment, and the people who surround them (Grinker et al., 1961; Beck and Bredemeier, 2016). The pandemic and its consequences —quarantine, social distancing, and self-isolation— increased loneliness and reduced social interactions, both of which are well-known risk factors for depression. Concerns about one’s own health and that of the beloved ones, along with the uncertainty about the future, can generate or exacerbate fear and depression (Fiorillo and Gorwood, 2020).

Anxiety is a complex emotional response and a fruit of the interaction between individual factors and specific situations (Endler and Parker, 1992). It is expressed through a varying pattern of cognitive, physiological, and motor responses (Lang, 1968). Many specialists agree that experiencing low levels of anxiety is normal and even advisable because the processes that anxiety awakens in the central and peripheral nervous system keep the person alert to face any situation and prone to give an immediate response. However, the problem arises when anxiety is generalized, particularly when it becomes a daily part of a person’s life and prevents them from feeling and doing things in a normal way. Apart from the predisposition, anxiety can increase when people face intense situations, strong traumas, or events that surpass their will or the resources to face them, as what happened to healthcare professionals during the COVID-19 pandemic (Weibelzahl et al., 2021).

Intolerance of uncertainty has a strong impact on mental wellbeing in a pandemic setting. This is significantly mediated by rumination and fear (Satici et al., 2020; Weibelzahl et al., 2021). In its simplest form, uncertainty is a “psychological state of ‘not knowing”’ (Kuang, 2017). More specifically, intolerance of uncertainty refers to the tendency to experience situations in which the result is yet unknown (but it is potentially known in the fullness of time as deeply aversive), independently from the valence of the result (Freeston et al., 1994). For Freeston et al. (2020), the coronavirus (COVID-19) is a new disease and an unprecedented challenge for healthcare workers and contemporary society in the broadest sense. Uncertainty distress, defined as “the subjective negative emotions experienced in response to the aspects of a given situation that are yet unknown,” is real and understandable, and the current methods of anxiety can only partially explain the level and the extent of the experienced anxiety. Rather than pathologizing anxiety in the context of a pandemic (Freeston et al., 1994, 2020) propose the concept of uncertainty distress as a normalizing model since it allows the understanding of the variety of factors they are dealing with and how anguish would be a reaction that evidence a normal response to an abnormal experience.

Finally, from a cognitive-behavioral perspective, coping consists of “constantly changing cognitive and behavioral efforts to manage external and/or internal demands that are appraised as taxing or exceeding the resources of a person” (Lazarus and Folkman, 1984, p. 141). Coping strategies point to dealing directly with the stressor (coping centered on the problem) or to regulating the emotions that emerge as a consequence of the stressful encounter (coping centered on the emotions) (Lazarus and Folkman, 1984). Moos and Billings (1982) added to the two general dimensions of Lazarus and Folkman the dimension of coping centered on the assessment. In general terms, coping centered on the emotion is considered to be dysfunctional and ineffective, while the less consistent effects, although generally positive, have been associated with coping centered on the problem (Folkman and Moskowitz, 2004; Taylor and Stanton, 2007; O’Driscoll et al., 2009). Boyd et al. (2009) also proved that coping centered on the emotion was associated with adverse results, such as increase of anxiety, emotional exhaustion, and dissatisfaction, while coping centered on the problem was associated with less emotional exhaustion. According to Folkman and Moskowitz (2004), the need for coping emerges in intensely emotional contexts, and an initial function of coping “is to downregulate negative emotions that are stressful in and of themselves and maybe interfering with instrumental forms of coping” (p. 747). The short-term adoption of coping strategies centered on the emotion may therefore be adaptive when the stressors are evaluated as uncontrollable and when there are insufficient resources, which allow people to amalgamate the necessary resources to participate in future coping strategies focused on the problem (Terry, 1994; Ben-Zur, 2009). Nonetheless, the sole and persistent dependency on coping strategies centered on the emotion or on strategies of avoidance for long periods is not considered beneficial. Behaviors of coping centered on the emotion encourage the person to disconnect from the problem, which prevents new attempts to face it and minimally contributes to directly approaching the stressor (Semmer, 2006; Ben-Zur, 2009). However, Lazarus and Folkman (1984) suggest that no coping strategy is intrinsically efficient or inefficient. Instead, the effectiveness of a given coping strategy depends on how well it corresponds with the evaluations and the situational conditions (Cummings and Cooper, 1998; Folkman and Moskowitz, 2004; Dewe and Cooper, 2007). Therefore, the crucial components that determine the effectiveness of coping are the adjustment and the context (Biggs et al., 2017).

In this unprecedented crisis of COVID-19 pandemic, healthcare workers are a special group of risk, facing infected patients, being exposed to a context of unpredictable future, and potentially suffering all the psychosocial effects mentioned above in several degrees. There is evidence that shows that the healthcare workers involved in the treatment of patients with COVID-19 develop a series of perceived concerns and threats, such as the fear of contagion, of infecting their loved ones, of making wrong decisions due to sleep deprivation, of having to decide whom to attend and who not to attend, among others, which affect their psychological wellbeing (Lai et al., 2020; Richaud et al., 2021a,b). In an initial study performed during the third week of mandatory quarantine in Argentina and based on the answers of 809 members of the healthcare personnel dedicated to the patients with COVID-19, higher values in the indicators of depression, anxiety, intolerance of uncertainty, and development of dysfunctional coping strategies were observed (Richaud et al., 2021b).

Xiao et al. (2020) also conducted an observational study during the current COVID-19 pandemic with 180 health workers who provided direct assistance to patients with COVID-19 and found significant levels of anxiety and stress which had a negative influence on the worker’s quality of sleep and self-efficacy (Xiao et al., 2020). It is important to highlight that in this study, those who informed to have a strong social support network had a lower level of stress and anxiety and had a higher level of self-efficacy. In another study that assessed the impact on mental health and the perceptions of psychological attention among medical and nursing personnel in China during the COVID-19 pandemic (Kang et al., 2020), a rise in the levels of psychological distress was detected, with both the exposure to infected people and the need of psychological assistance being identified as related factors. Although these healthcare workers had access to mental health facilities, though in a limited way, the personnel under distress identified these as important resources to alleviate acute disorders of mental health and improve their perceptions of physical health.

On the other hand, Leung et al. (2005) indicate that in studies performed in previous epidemics, the stability and temporary evolution of the psycho-behavioral responses to an outbreak remained undefined due to the exclusively transversal nature of those studies. Lee et al. (2007) provided information about the potential long-term negative psychological effects of infectious diseases. Based on the impact of the 2003 Severe Acute Respiratory Syndrome (SARS) outbreak, their results showed that instead of decreasing with time, the stress levels were consistently higher a year after the outbreak. The psychological state of the health personnel involved in the caring of patients with SARS was particularly worrying given its alteration in all the measures of stress and psychological distress as compared to the other non-health professional workers who survived the SARS. At the same time, Chan and Huak (2004) studied the psychological impact of SARS in 661 health workers of a regional hospital 2 months after the outbreak and found that 20% of all the participants presented indicators of post-traumatic stress and that many of them were still emotionally damaged and traumatized by the SARS outbreak.

Another aspect to take into account that could be affected by the passing of time and the perceived concerns and threats mentioned before, i.e., the increased values in the indicators of depression, anxiety, intolerance of uncertainty, and the use of dysfunctional coping strategies (Richaud et al., 2021b) is how it affects the worker’s quality of life (QoL). Woon et al. (2021) found that COVID-19-related factors (e.g., stress from loss of daily routine and stress due to annual leave being frozen) and psychological complications (greater severity of depression and stress symptoms) contributed to the lowering of psychological QoL in accordance with previous studies (Çelmeçe and Menekay, 2020; Suryavanshi et al., 2020). Specifically, COVID-19 induced social functional impairment that is strongly associated with depression and poor psychological wellbeing (Dawel et al., 2020). This means that the greater severity of depression and stress predicted lower social relationship QoL (Dawel et al., 2020; Vafaei et al., 2020; Woon et al., 2021). In the opposite manner, QoL among healthcare workers was greater with the higher perceived social support received from friends and significant others (Woon et al., 2021).

With these records, it was considered to be important to analyze how the concerns and the indicators of mental health were different among the health personnel throughout the development of the pandemic in our country.

Therefore, the objective of the present study is to analyze the difference in the psychological distress of healthcare workers in three different stages of the COVID-19 pandemic (first period: April 7–14, second period: April 15–22, third period: April 23–30 of 2020, each lasting 24 days). These periods started after the third week of mandatory quarantine in Argentina, and in each of them, the level of exposure of the health personnel to a larger number of patients under treatment for COVID-19 increased. During this period of time, for several months, the mandatory lockdown of the whole country was absolute. It was forbidden to go out on the street except for basic purchases in nearby stores and the use of public transportation was only for essential workers. This aggravated the already poor economical situation, causing some sectors of the population to not have the means to afford basic necessities of life, such as food. In the meantime, the number of cases was increasing along with the death rates, although in a slower manner compared to other parts of the world. At this point, there were no specific treatments or vaccines for the virus. Hence, the fear of not having enough available hospitals, equipment, and healthcare workers to respond to the high demand was rising.

## Materials and Methods

### Participants

The 1,458 participants of the sample were health personnel (doctors, nurses, physical therapists, biochemists, etc.) involved in the care of patients with COVID-19 from the 32 hospitals of the country. The participants were distributed according to the following regions and provinces: Cuyo (Mendoza, San Juan, San Luis), 115 (7.89%); North (NOA-NEA: Tucumán, Salta, Misiones, Chaco, Corrientes, Santiago del Estero, Jujuy, Formosa, Catamarca, La Rioja), 355 (24.35%); Center (Córdoba, Santa Fe, Entre Ríos), 378 (25.93%); Patagonia (Río Negro, Neuquén, Chubut, La Pampa, Santa Cruz, Tierra del Fuego), 91 (6.24%); and Buenos Aires (AMBA: province of Buenos Aires, Buenos Aires City), 519 (35.6%).

The data were collected through a digitalized questionnaire that was distributed through the online survey tool SurveyHero. We established contact with different health entities of the Argentine government, which allowed access to hospitals in the different provinces of the country. In addition, contact was made with directors of health centers in addition to the use of social networks such as health personnel Facebook groups, Twitter, Instagram, and WhatsApp. This was done to ensure a wider reach within the different provinces that integrate the Argentine Republic. In the cover of the questionnaire, a statement of agreement with an informed consent that was included was placed as a mandatory field to be completed. To protect the privacy of the subjects, the survey was conducted anonymously. The instruction specified that only healthcare workers dedicated to the treatment of patients with COVID-19 responded. In all cases, those patients were treated in isolated areas, ensuring that the health personnel did not treat patients with other pathologies. The questionnaire was answered by 1,458 healthcare workers, 1,159 (79.5%) of which are women and 299 are men, with a mean age of 41.58 (*SD* = 10.41). From the sample, 64.4% worked in state facilities, and 35.6% worked in private institutions. In addition, 630 people (43.2%) worked in the emergency room (*n* = 218, 15.0%); general hospitalization (*n* = 255, 17.5%); intensive care unit (ICU; *n* = 133, 9.1%); and 56.8% in other areas (kinesiology, radiology, laboratory, and ambulance).

The answers of the sample were divided into three periods of 8 days each on April 2020, which encompassed 38.4% of the participants in the first period (April 7–14), 25.4% in the second period (April 15–22), and 36.2% in the third period (April 23–30), respectively. It is important to highlight that this design includes three cross-sectional studies in three independent samples (1, 2, 3) with the following characteristics: size (Period 1: *n* = 560; Period 2: *n* = 370; Period 3: *n* = 528), age (Period 1: *M*_age_ = 42.99; Period 2: *M*_age_ = 42.96; Period 3: *M*_age_ = 39.88), and gender (Period 1: female = 81.4%; Period 2: female = 74.1%; Period 3: female = 81.9%). It was impossible to carry out a longitudinal study due to anonymous participation. Given the sensitive pandemic context in which the assessment was carried out, there was a risk that participants would fear being identified and judged negatively.

### Instruments

A *questionnaire* was created with three sections (Richaud et al., 2021b):

1) Sociodemographic data.
2) 20 questions related to the concerns of the health personnel regarding the coping of patients with coronavirus extracted from the preliminary interviews and statements given by the health personnel. Due to the pandemic, the interviews were conducted through video calls. We inquired about the main concerns that health professionals had about the pandemic. Subsequently, the responses were transcribed, analyzed, and categorized by 6 expert psychologists. Then, those that had appeared more frequently among the participants were selected. Some of these stressors matched others mentioned in preceding studies (Tan et al., 2020; Windarwati et al., 2021).

The selected questions were as follows:

a) Answered Never/Almost never, Rarely, Often, Always/Almost always:
Are you worried about the possibility of being infected by COVID-19?
Are you worried about the possibility of infecting your loved ones?
Do you feel stigmatized?
Do you fear having to decide at some point whom to attend and who not to attend?
If so, do you participate in one?
b) Answered Yes, No:
Does exhaustion interfere with your work?
Did the work environment change with the onset of the pandemic?
If it changed, did it worsen?
Is there a group of support for the health personnel at your workplace?
Do you believe that counting on mental health personnel who supports you would help you cope with your concerns?
Do you have adequate equipment?

Following the guidelines proposed by the World Health Organization (2020), “adequate equipment” is considered to be personal protective equipment that constitutes the most effective preventive measure as a strategy to avoid the transmission of COVID-19. This equipment consists of the following supplies: medical and self-filtering masks, medical gowns, eye-protection glasses, face shields, and gloves (World Health Organization, 2020).

3) Questions that referred to depression, anxiety, intolerance of uncertainty, and coping were taken from the short versions (Richaud et al., 2021b) of: (a) the Argentine adaptation of the Beck Depression Inventory (BDI) Questionnaire (Richaud de Minzi and Sacchi, 2001a,b) the Argentine adaptation of the Anxiety Traits and Situations Inventory (ISRA) (Richaud de Minzi and Sacchi, 1995); (c) the Argentine adaptation of the Intolerance of Uncertainty test (IUS) (Rodríguez, de Behrends and Brenlla, 2015); and (d) the Argentine adaptation of the Ways of Coping Questionnaire (WCQ) (Richaud de Minzi and Sacchi, 2001b). The questions regarding coping specifically referred to the stressor of caring for patients with COVID-19. All the items were presented to be answered using a Likert scale of 4 points, with (1) being Almost never/Never, and (4) being Almost always/Always (Richaud et al., 2021b).

The Cronbach alphas for this study samples were the following: depression, 0.70; anxiety, 0.82; intolerance of uncertainty, 0.80; and coping, 0.70.

### Ethical Procedure

The project and questionnaire had the endorsement of the Research Ethics Committee of the Faculty of Health Sciences of the Adventist University of Plata, with No. CE000237 of the National Registry of Research in Health and N° 3999 of Ministerial Resolution of the Ministry of Health of the Province of Entre Ríos, Argentina, Resolution 1.4/2020.

The informed consent was approved by the Research Ethics Committee, created by the ministerial resolution 1002/16 and by the Personal Data Protection Law 25.326.

### Statistical Analysis

The following descriptive measures were calculated: percentages, arithmetic means, standard deviations, skewness, and kurtosis. Questions regarding concerns and fears were re-categorized into Yes (Never/Rarely, Few times) and No (Always/Almost always, Many times). Chi-square (*X*^2^) tests were carried out to study the association between the period and the different fears and worries. Multivariate ANOVAs (MANOVAs) for non-repeated measures were conducted (*F*Hotelling for the general differences and univariate *F* for the differences in each variable) to analyze the influence of the different concerns in the indicators of depression, anxiety, intolerance of uncertainty, and coping. For all the statistical calculations, the SPSS.24 statistical package was used.

## Results

### Preliminary Analysis

The skewness and kurtosis values did not exceed the numbers of ± 1.5 recommended for parametric analysis in any variable (Muthen and Kaplan, 1992; Forero et al., 2009). First, we had to analyze whether there was a difference in the indicators of depression, anxiety, intolerance of uncertainty, and coping among health professionals who worked in different areas (emergency room, general hospitalization, ICU, kinesiology, radiology, laboratory, and ambulances) while controlling all three periods. Results indicated that there were no differences between the indicators based on the work area of the health professionals included in the study [indicators of depression, *F*Hotelling (16, 5,766) = 1.47, *p* = 0.10; indicators of anxiety, *F*Hotelling (24, 5,762) = 1.18, *p* = 0.25; indicators of intolerance of uncertainty, *F*Hotelling (12, 4,431) = 1.13, *p* = 0.33; and indicators of coping, *F*Hotelling (32, 5,698) = 0.92, *p* = 0.60]. Since no statistically significant differences were found, successive analyzes were carried out with the total study sample without discriminating the work area.

### Main Results

The obtained results are presented by drawing from an analysis of the responses that were considered more relevant to the objectives of the study and the indicators of depression, anxiety, intolerance of uncertainty, and coping, according to each period, throughout the time of recording.

#### Fear of Being Infected by the COVID-19

The fear of getting infected was significantly associated with each period [*X*^2^(2) = 9.33; *p* = 0.009], especially in the third period (Table 1), as shown by values going from the 64.5% of participants in the first period to the 71% in the third one.

**TABLE 1:** Relationship between period and concerns, interference of exhaustion at work, change in work environment, and existence of a psychological support group.

	Period 1		Period 2		Period 3		Total		
	*n*	(%)	*n*	(%)	*n*	(%)	*n*	(%)	*p*
**Fear of contagion**									
Yes	360	(64.5)	225	(61.1)	372	(70.6)	957	(65.9)	
No	198	(35.5)	143	(38.9)	155	(29.4)	496	(34.1)	
Total	558	(100)	368	(100)	527	(100)	1,453	(100)	**0.009**
**Fear of infecting**									
Yes	464	(83.2)	312	(84.8)	474	(89.9)	1,250	(86.0)	
No	94	(16.8)	56	(15.2)	53	(10.01)	203	(14.0)	
Total	558	(100)	368	(100)	527	(100)	1,453	(100)	**0.004**
**Protection equipment**									
Yes	196	(38.4)	131	(41.7)	209	(39.3)	536	(36.9)	
No	362	(64.9)	236	(64.3)	318	(60.3)	916	(63.19)	
Total	558	(100)	367	(100)	527	(100)	1,452	(100)	0.259
**Stigmatization**									
Yes	76	(13.6)	55	(15.0)	87	(16.4)	218	(15.0)	
No	483	(86.4)	312	(85.0)	442	(83.6)	1,237	(85.4)	
Total	559	(100)	367	(100)	529	(100)	1,455	(100)	0.420
**Fear of decision**									
Yes	204	(36.6)	123	(33.6)	203	(38.7)	530	(36.6)	
No	354	(63.4)	243	(66.4)	322	(61.3)	919	(63.4)	
Total	558	(100)	366	(100)	525	(100)	1,449	(100)	0.304
**Exhaustion at work**									
Yes	373	(66.8)	263	(71.3)	418	(79.2)	1,054	(72.4)	
No	185	(33.2)	106	(28.7)	110	(20.8)	401	(27.6)	
Total	558	(100)	369	(100)	528	(100)	1,455	(100)	**0.000**
**Change in work environment**									
Yes	511	(91.1)	345	(93.2)	511	(96.4)	1,367	(93.6)	
No	50	(8.9)	25	(6.8)	19	(3.6)	94	(6.4)	
Total	561	(100)	370	(100)	530	(100)	1,461	(100)	**0.002**
**Way of change**									
Got worse	379	(67.6)	266	(71.9)	421	(79.4)	1,066	(80.5)	
Got better	130	(23.2)	71	(19.2)	84	(15.8)	285	(19.5)	
Total	561	(100)	370	(100)	530	(100)	1,461	(100)	**0.000**
**Support to the health workers**									
Yes	167	(29.9)	103	(28.0)	188	(35.7)	458	(31.5)	
No	391	(70.1)	265	(72.0)	338	(64.3)	994	(68.5)	
Total	558	(100)	368	(100)	526	(100)	1,452	(100)	**0.009**
**Participation in a support group**									
Yes	65	(23.6)	52	(30.6)	49	(19.4)	166	(23.8)	
No	210	(76.4)	118	(69.4)	204	(80.6)	531	(76.2)	
Total	275	(100)	170	(100)	253	(100)	697	(100)	**0.029**
**Belief in the help of a support group**									
Yes	404	(77.8)	257	(77.6)	378	(79.4)	1,039	(78.4)	
No	115	(22.2)	74	(22.4)	98	(20.6)	287	(21.6)	
Total	519	(100)	331	(100)	476	(100)	1,326	(100)	0.797

*Bold values highlight significant differences between periods.*

#### Concern About Infecting Family and Friends

The fear of infecting their loved ones showed significant association with each period [*X*^2^(2) = 11.03; *p* = 0.004], especially during the third period (Table 1), with values going from 83 to 90% of the participants.

#### Availability of Adequate Equipment

Table 1 also shows that 63.2% of the participants answered that they did not have the appropriate equipment. It is observed that this percentage remains similar throughout the three periods [*X*^2^(2) = 2.71; *p* = 0.26], although it somehow decreases in the third period.

#### Perception of Stigmatization

As seen in Table 1, only 15% of the participants perceived stigmatization. This percentage remains similar throughout the three periods [*X*^2^(2) = 1.75; *p* = 0.42].

#### Fear of Having to Decide Who to Attend and Who Not to Attend

Table 1 also shows that 37% of the participants expressed fear of having to decide who to attend and who not to attend. This percentage remains unchanged throughout the three periods [*X*^2^(2) = 2.38; *p* = 0.30].

#### Interference of Exhaustion at Work

It is observed that 72.4% of the participants expressed interference from exhaustion at work, with a significant difference [*X*^2^(2) = 20.96; *p* = 0.001] ranging from 66.8% in the first period to 79.2% in the third (Table 1).

#### Perception of Differences in the Work Environment

As seen in Table 1, 93.6% of the participants perceived differences in the work environment with a significant association to the three periods [*X*^2^(2) = 12.20; *p* = 0.002], as shown by values reaching 96.4% in the last period.

#### Perception of Worsening of Work Environment

Table 1 also shows that 80% of the participants perceived that their work environment worsened. In addition, it is observed that the percentage associated to the worsening had a significant difference [*X*^2^(4) = 21.64; *p* = 0.001], with values increasing from 67.6% in the first period to 79.4% in the third.

#### Existence of and Participation in a Psychological Support Group in the Workplace

As shown in Table 1, 68.5% of the participants expressed no support or containment group for the health personnel at their workplace. At the same time, it is observed that there was a significant difference in the existence of psychological support groups [*X*^2^(2) = 13.55; *p* = 0.01], especially between the first and third periods.

In the question “**If so, do you participate in one?”** only 24% said that they do it generally, while, in turn, a significant difference of this involvement is observed, going from 24 to 19% as time advances. Although there had been an increase in the number of available groups of psychological support, in the different groups corresponding to each period, the involvement in those support groups significantly decreases [*X*^2^(2) = 7.06; *p* = 0.03] (Table 1). At the same time, given that this 24% refers to the 32% who answered that they have a support group, only 8% of the total sample participates in these groups.

When asked “**If you do not receive any support, do you believe that counting on mental health personnel (psychologist, psychiatrist) who listens to you and supports you would help you cope with your concerns?,”** 78% answered positively, without showing significant differences over the periods [*X*^2^(2) = 0.49; *p* = 0.78] (Table 1). Once again, it seems curious that if 78% believe they need support, only 8% are receiving said help.

### Differences in the Indicators of Depression, Anxiety, Intolerance of Uncertainty, and Coping Strategies Between Groups of Healthcare Workers in the Three Stages

#### Depression

As the time of exposure advanced, there were differences in all the indicators of depression [*F*Hotelling (8, 2,896) = 14.62; *p* **<** 0.001], especially between the groups of the first and the third period and between those of the second and the third, with increased values in *I am more irritated than before* and *I feel sad*. However, the most noticeable one is *I do not sleep as well as before*, which reaches a mean value of 2.85 in the third period (Table 2).

**TABLE 2:** Differences in indicators of depression, anxiety, intolerance of uncertainty, and coping between periods.

	Period 1		Period 2		Period 3		
Items	*M*	*SD*	*M*	*SD*	*M*	*SD*	*F*
**Depression**							
I am more irritated than before	2.19^a^	0.03	2.34^b^	0.04	2.54*^c^*	0.04	24.14***
I feel sad	2.34^a^	0.03	2.37^a^	0.04	2.64^b^	0.04	20.42***
I do not sleep as well as before	2.29^a^	0.04	2.45^a^	0.05	2.85^b^	0.04	47.41***
I feel guilty when I am resting	1.71^a^	0.04	1.82^a^	0.05	2.02^b^	0.04	15.46***
**Anxiety**							
I feel insecure	2.16^a^	0.04	2.29^a^	0.05	2.47^b^	0.04	16.69***
I feel scared	2.36^a^	0.04	2.32^a^	0.05	2.65^b^	0.04	20.92***
I feel discomfort in my stomach	1.87^a^	0.04	1.88^a^	0.05	2.27^b^	0.04	28.70***
My body is tense	2.51^a^	0.04	2.56^a^	0.05	2.97^b^	0.04	42.71***
I cry or moved easily	2.38^a^	0.04	2.40^a^	0.05	2.69^b^	0.04	15.97***
I move and do things without and end in themselves	1.87^a^	0.04	1.89^a^	0.05	2.13^b^	0.04	14.9***
**Intolerance of uncertainty**							
I cannot be at peace if I do not know what will happen tomorrow	2.17^a^	0.04	2.19^a^	0.05	2.5^b^	0.04	19.18***
Unexpected events bother me a lot	2.43^a^	0.04	2.51^a^	0.05	2.72^b^	0.04	15.10***
I feel that even with the best planning, a small detail could ruin it all	2.31^a^	0.04	2.38^a^	0.05	2.65^b^	0.04	19.62***
**Coping**							
I focus exclusively in what I have to do, step by step	3.31	0.03	3.34	0.04	3.38	0.03	1.19
I propose a different solution when the protocol fails	2.74^a^	0.03	2.91^b^	0.04	3.00^b*c*^	0.04	13.40***
I speak to someone who can help me when the situation overwhelms me	2.94	0.03	3.18	0.04	3.05	0.04	9.20***
I try to bring something positive out of the situation	3.15^a^	0.03	3.20^a^	0.04	3.01^b^	0.03	6.97**
I try not to think about what is happening	2.24^a^	0.04	2.28^a^	0.05	2.09^b^	0.04	5.85**
I accept it since there is nothing I can do about it	2.62^a^	0.04	2.62^a^	0.05	2.47^b^	0.04	4.72**
I burst out over anything	1.88^a^	0.04	1.99^a^	0.04	2.20^b^	0.04	19.83***
I try to control my emotions	3.05	0.04	3.07	0.03	3.02	0.04	0.60

The means with different subscripts indicate between which groups the significant differences are observed. (**p ≤ 0.01; ***p ≤ 0.001).

In terms of percentage, irritability goes from 34 to 53% [*X*^2^(2) = 40.68; *p* < 0.001] and sleep disorders from 43% in the group of the first period to 67% in that of the third [*X*^2^(2) = 65.34; *p* < 0.001] (Table 3).

**TABLE 3:** Relationship between period and percentage of irritability, sleep disorders, and body tension.

	Period 1		Period 2		Period 3		Total		
	*n*	(%)	*n*	(%)	*n*	(%)	*n*	(%)	*p*
**Irritability**									
Yes	192	(34.4)	148	(40.0)	282	(53.5)	622	(42.7)	
No	366	(65.6)	222	(60.0)	245	(46.5)	833	(57.3)	
Total	558	(100)	370	(100)	527	(100)	1,455	(100)	**0.002**
**Sleep disorders**									
Yes	238	(42.7)	182	(49.2)	352	(66.7)	772	(53.1)	
No	319	(35.5)	188	(38.9)	176	(33.3)	683	(46.09)	
Total	557	(100)	370	(100)	528	(100)	1,455	(100)	**0.000**
**Body tension**									
Yes	294	(52.7)	204	(55.3)	385	(72.9)	883	(60.7)	
No	264	(47.3)	165	(44.7)	143	(27.1)	572	(39.3)	
Total	558	(100)	369	(100)	528	(100)	1,455	(100)	**0.000**

*Bold values highlight significant differences between periods.*

#### Anxiety

In the case of anxiety, there was a significant difference in the value of all its indicators [*F*Hotelling (12, 2,894) = 9.17; *p* < 0.001], reaching scores that are especially high in the group of the third period for the indicators *I feel scared*, *I cry, or am moved easily* (lack of emotional control) and particularly *My body is tense* (alertness), which reaches a mean value of 2.97 (see Table 2) and, in terms of a percentage, goes from 53 to 73% [*X*^2^(2) = 52.58; *p* < 0.001] (Table 3).

#### Intolerance of Uncertainty

In the case of intolerance of uncertainty, as the time of exposure advanced, there was also a significant difference among the healthcare groups in all the indicators [*F*Hotelling (6, 2,900) = 8.94; *p* < 0.001], with the indicators *Unexpected circumstances bother me a lot* and *I feel that even with the best planning, a small detail could ruin it all* reaching especially high values (Table 2).

#### Coping Strategies

In the case of coping strategies for which the analysis is different, given that its functionality depends or not on the total profile of strategies, significant differences were also found [*F*Hotelling (16, 2,862) = 6.92; *p* = 0.001]. It is observed that the strategy *I focus exclusively on what I have to do, step by step*, has kept increased but constant values throughout the periods. A similar trend was also observed in the strategy *I propose a different solution when the protocol fails*, which had significantly different values in the three periods, reaching a maximum value in the third period. This indicated an exclusive focus on solving the problem concerning their job. At the same time, a significant difference in the strategy *I try to bring something positive out of the situation* was observed. Furthermore, there was a significant difference between the three groups in *I burst out over anything* (lack of emotional control), which had been observed in one of the indicators of anxiety and a very high level of emotional control, which remained the same throughout the three periods (Table 2).

### Relationship Between Gender and the Indicators of Depression, Anxiety, Intolerance of Uncertainty, and Coping Strategies Throughout the Three Periods Assessed

Since there were more women (*n* = 1,159) than men (*n* = 294), a subsample was randomly extracted from the sample of women. In each period and in proportion to the sample of men, three subsamples of women were extracted: Period 1 *N*males = 103, *N*females = 193; Period 2 *N*males = 95, *N*females = 128; Period 3 *N*males = 95, *N*females = 126. The total *N* of the subsample of females was 447 so that the size of the women sample and the men sample would be similar. From the comparison by gender, it shows that women obtained, in general, significantly higher values than men in all indicators of depression [*F*Hotelling gender (8, 1,458) = 4.06; *p* < 0.001; *F*Hotelling period (8, 1,458) = 5.42; *p* < 0.001]. However, in the case of *I feel sad*, women obtained significantly higher values than men in the first period. Despite this, this distance became smaller until it disappeared in the third period [*F*Hotelling (12, 2,186) = 1.82; *p* < 0.040], in which men obtained a slightly higher value than women (Table 4).

**TABLE 4:** Differences in indicators of depression, anxiety, intolerance to uncertainty, and coping according to gender and period.

	Period 1				Period 2				Period 3					
Variables	*M* Female	*SD* Female	*M* Male	*SD* Male	M Female	*SD* Female	*M* Male	*SD* Male	*M* Female	*SD* Female	*M* Male	*SD* Female	F gender (3,726)	F period (2,726)
**Depression**														
I am more irritated than before	2.27	0.06	1.87	0.08	2.33	0.07	2.27	0.08	2.44	0.07	1.89	0.08	2.55	12.12***
I feel sad	2.42	0.06	1.89	0.08	2.42	0.07	2.09	0.08	2.50	0.07	2.37	0.09	13.71***	4.71**
I do not sleep as well as before	2.33	0.07	2.01	0.09	2.41	0.08	2.36	0.10	2.75	0.09	2.55	0.10	4.05*	12.05***
I feel guilty when I am resting	1.73	0.06	1.42	0.09	1.82	0.08	1.65	0.09	1.97	0.08	1.81	0.09	5.12**	6.32**
**Anxiety**														
I feel insecure	2.20	0.07	1.81	0.09	2.44	0.08	1.93	0.09	2.40	0.08	2.16	0.09	11.40***	5.27**
I feel scared	2.46	0.06	2.03	0.09	2.40	0.08	1.96	0.09	2.54	0.08	2.27	0.09	11.72***	2.83*
I feel discomfort in my stomach	1.92	0.07	1.57	0.09	1.98	0.08	1.67	0.09	2.36	0.08	1.86	0.09	11.34***	5.12**
My body is tense	2.59	0.07	2.09	0.09	2.62	0.08	2.41	0.09	2.87	0.08	2.55	0.09	10.22***	8.31***
I cry or moved easily	2.49	0.07	1.55	0.09	2.57	0.08	1.97	0.09	2.70	0.08	1.91	0.09	42.01***	7.65**
I move and do things without and end in themselves	1.89	0.06	1.60	0.08	1.96	0.08	1.74	0.09	2.20	0.08	1.95	0.09	6.12***	6.04**
**Intolerance of uncertainty**														
I cannot be at peace if I do not know what will happen tomorrow	2.17	0.07	1.92	0.09	2.26	0.08	1.97	0.10	2.40	0.08	2.10	0.10	8.47***	1.870
Unexpected events bother me a lot	2.44	0.07	2.29	0.09	2.50	0.08	2.48	0.10	2.60	0.08	2.47	0.10	1.11	3.138*
I feel that even with the best planning, a small detail could ruin it all	2.31	0.07	2.11	0.09	2.37	0.08	2.28	0.10	2.58	0.08	2.47	0.10	1.74	5.062**
**Coping**														
I focus exclusively in what I have to do, step by step	3.31	0.06	3.32	0.07	3.35	0.07	3.36	0.08	3.36	0.07	3.38	0.08	3.57*	0.48
I propose a different solution when the protocol fails	2.74	0.06	2.75	0.08	2.96	0.08	2.86	0.09	2.94	0.08	2.96	0.09	0.45	4.06*
I speak to someone who can help me when the situation overwhelms me	2.89	0.06	2.84	0.08	3.26	0.07	3.09	0.09	2.98	0.08	3.09	0.09	3.19*	5.35**
I try to bring something positive out of the situation	3.04	0.06	3.28	0.08	3.17	0.07	3.23	0.08	3.07	0.07	3.05	0.08	1.76	0.37
I try not to think about what is happening	2.22	0.07	2.21	0.09	2.28	0.08	2.22	0.09	2.09	0.08	2.05	0.09	2.17	0.81
I accept it since there is nothing I can do about it	2.22	0.07	2.21	0.09	2.28	0.08	2.22	0.09	2.09	0.08	2.05	0.09	2.17	0.81
I burst out over anything	2.62	0.08	2.59	0.09	2.62	0.08	2.63	0.09	2.40	0.08	2.52	0.09	1.21	0.11
I try to control my emotions	2.96	0.06	3.10	0.08	3.14	0.07	3.11	0.08	2.98	0.07	3.12	0.08	3.19*	0.47

Multivariated Analysis Depression: Period F_Hoteling_ (8, 1,460) = 5.42; p < 0.001, Gender F_Hoteling_ (8, 1,458) = 4.06; p < 0.001, Gender by period F_Hoteling_ (12, 2,186) = 1.82; p = 0.040; Multivariated Analysis Anxiety: Period F_Hoteling_ (12, 1,454) = 4.33; p < 0.001, Gender F_Hoteling_ (18, 2,180) = 7.94; p < 0.001, Gender by Period F_Hoteling_ (18, 2,180) = 1.60; p = 0.052; Multivariated Analysis Intolerance of uncertainty: Period F_Hoteling_ (6, 1,460) = 1.93; p = 0.072, Gender F_Hoteling_ (6, 1,460) = 3.001; p = 0.006, Gender by Period F_Hoteling_ (9, 2,189) = 473; p = 0.893; Multivariated Analysis Coping: Period F_Hoteling_ (16, 1,436) = 1.64; p = 0.052, Gender F_Hoteling_ (24, 2,153) = 3.63; p < 0.001, Gender by Period F_Hoteling_ (24, 2,153) = 0.565; p = 0.956. (*p ≤ 0.05; **p ≤ 0.01; ***p ≤ 0.001).

Regarding **anxiety**, women showed values that were significantly higher than in men [*F*Hotelling (18, 2,180) = 7.96; *p* **<** 0.001], but over time, the indicators of anxiety showed significant differences both for women and men [*F*Hotelling (12, 1,454) = 4.33; *p* **<** 0.001]. Particularly, the statement *My body is tense* reached especially high values in the third period in both genders. On the other hand, the statement *I cry or am moved easily* also reached high values in women in the third period [*F*Hotelling (18, 2,180) = 1.82; *p* **<** 0.052] (Table 4).

Regarding **intolerance of uncertainty**, as compared to men, women obtained higher values in all the indicators [*F*Hotelling (6, 1,460) = 3.00; *p* **<** 0.006] in all periods [*F*Hotelling (6, 1,460) = 1.93; *p* **<** 0.072] (Table 4). In this case, the values also reached high scores over time in both genders, with the statement *Unexpected events bother me a lot* and *I feel that even with the best planning a small unexpected event might ruin it all* being especially higher in the third period.

Finally, regarding **coping**, the most important differences were found in the statement *I try to control my emotions*, in which men obtained significantly higher values than women, and *I burst over anything*, where women obtained higher values. Strict control of emotions was found to be coping strategy that was most frequently used, although it was somehow weaker in women, who, according to the data, lost it more easily [*F*Hotelling (16, 1,436) = 1.64; *p* **<** 0.050; *F*Hotelling (24, 2,153) = 3.63; *p* **<** 0.001] (Table 4).

## Discussion

As mentioned above, in a previous study we conducted during the third week of the mandatory quarantine, a preliminary diagnosis was carried out regarding how affected the psychological wellbeing of the health personnel dedicated to the attention of patients with COVID-19 was (Richaud et al., 2021b). In all the cases, it was found that health personnel dedicated to the treatment of patients with COVID-19 presented higher values in the rates of ***depression***, ***anxiety*,** and ***intolerance of uncertainty*** and informed ***dysfunctional coping strategies***, whether through lack of control or avoidance.

The present study analyzed the difference in the psychological situation of three groups of healthcare workers from the third week of the mandatory quarantine in Argentina (first group/period) and through the two following weeks. Based on the responses of 1,458 health workers in public and private environments from the entire country, from different professions, and attending in various areas, the differences in the indicators of psychological distress were analyzed corresponding to what these workers mentioned during the 3-week period. The **main conclusions** are the following:

**1-**Regarding the **threats to the psychological wellbeing** of the health personnel involved in the attention of patients with coronavirus, the principal concern was the possibility of infecting their loved ones, followed by the concern of infecting themselves, followed in turn by the possibility of having to decide who to attend and who not to attend. In general terms, these results concerning the main threats perceived by health personnel coincide with those of other studies carried out in relation to the SARS pandemic in 2003 and to the COVID-19 pandemic (Maunder et al., 2003; Marjanovic et al., 2007; Liu et al., 2012; Lai et al., 2020) although they did not analyze the differences in the perception of the threat at different moments in time. Only few healthcare workers mentioned feeling stigmatized. In addition, there were non-significant differences through time. This was reflected in accounts such as below:

*I do not fear for myself, but for my family. I went to my parents’ farm, my wife is asthmatic, but I want the spike to be over so I do not infect her (Administrative employee, Autonomous City of Buenos Aires Hospital).*

*I fear contagion and being intubated… and, logically, death (Intern Medicine specialist in the Autonomous City of Buenos Aires Public Hospital and PAMI; Comprehensive Medical Attention Program, a public health insurance agency for the elderly managed by the Ministry of Health).*

It should be noted that this health personnel has not felt stigmatized, contrary to what was reported by other authors who indicated that stigmatization was an important aspect highlighted by healthcare workers (Maunder et al., 2003; Brooks et al., 2020; Wu et al., 2020). Moreover, according to Brooks et al. (2020), “stigma from others” persisted even after the quarantine, and healthcare workers felt more stigmatization than the general public.

**2-**Regarding their **perception of how they are being taken care of**, it was found that in general, a high percentage of healthcare workers considered that they did not have the appropriate equipment. This remained similar in the three groups, although it was slightly lower in the last period. This was evident in accounts such as:

*The lack of supplies was the first that struck us; we had surgical masks that generally last over 2–3 h, and we were on call 10 h with only one face mask (Head nurse, Mendoza Public Hospital).*

In this regard, it should be noted that existing literature has shown that the fear of lack of appropriate equipment greatly increased anxiety among healthcare workers during the COVID-19 pandemic (Woon et al., 2020).

The **perception of how the work environment worsened** was significantly different among the groups. It increased from the first to the third group. The perception that sleep deprivation interfered with their work reached a very high percentage compared with the values reported by other authors (Xia et al., 2021). This was reflected in the following account:

*This past week they tried to divide us into teams, and that is when the personnel who was working simultaneously was reduced and I had much more work to do. I am on call every other day, and I have three night shifts. It is a lot of stress and exhaustion (Intern doctor in Entre Rios).*

**3-**The chance of **counting on a psychological support team** was low. Despite this, surprisingly, among those who mentioned having these teams, the participation was significantly lower when comparing the first group/period with the third group/period. Finally, most healthcare workers said that having a support group would help with their problems and fears. Statistically, this did not have significant differences among the groups/periods. This was described by a nurse in Mendoza as follows:

*The truth is we are not used to using technology for this. We are not allowed to express ourselves, nor can we expand on what we are feeling at the moment. So much of this leads to failure of that intervention.*

**4-**Due to the fact that the first report showed that **indicators of depression, anxiety, and intolerance of uncertainty** were significantly affected by the concerns manifested by the health personnel (Richaud et al., 2021b) and that the studies carried out in three groups in different periods of time indicated that many of the concerns had increased through the analyzed periods while the psychological help remains in very low levels, it is concluded that the psychological indicators have also been drastically modified.

All had significantly higher values from the first group/period to the third group/period with regard to the indicators of depression. This was particularly observed in the statement *I feel more irritated than before*, *I feel sad* and in the statement *I do not sleep as well as before*. Noticeably, irritability and sleep disorders significantly increased from the first to the third group/period, surpassing the values of insomnia shown by Lai et al. (2020) and Zhang et al. (2020) in the samples of health personnel involved in the treatment of patients with COVID-19 in China. Regarding irritability, in the present study, it was observed (with some surprise) that its first records showed relatively low values, which were in contrast with some public manifestations of the health personnel (collected during the week of April 20, 2020 through media). The current results show there has been a significant difference in irritability, especially between the second and the third groups/periods.

All values of the indicators of anxiety have significantly increased from the first to the third group/period, especially in the statement *I feel scared*, *I cry or am moved easily* (lack of emotional control) and particularly *My body is tense* (alertness). Also, there were significant differences between the three groups/periods in the indicators of intolerance of uncertainty. This was especially observed in the statements *Unexpected events bother me a lot* and *I feel that even with the best planning, a small unexpected event might ruin it all*. In this regard, when studying health personnel during the SARS pandemic of 2003, Maunder et al. (2003) found an increased perception of personal danger due to uncertainty generated by the constant amendment of the procedures and the public health guidelines to control/prevent infection. The same was observed by Di Monte et al. (2020) who pointed out that the impact the COVID-19 emergency had on doctors was partly produced by the uncertainty of the necessary procedures and treatments, along with the immediate saturation of hospitals for the management of critical cases. They observed avoidance of uncertainty and paralysis when it appeared. Furthermore, in reference to the increased values of anxiety, depression, and irritability, these have also been observed by other researchers (e.g., Neto et al., 2020; Wu et al., 2020).

These differences in the indicators of high tension and in the lack of emotional control were also expressed in the coping strategies.

The ways of coping with conflict had differences between the three groups/periods of time. The values for the items *I try to bring something positive out of the situation*, *I try not to think about what is happening*, and *I accept it since there is nothing I can do about it* significantly decreased, indicating less avoidance and less cognitive resignification which would allow for a greater flexibility in the response to the threat by restructuring and turning it into something more manageable. At the same time, *I burst out over anything* increased significantly which, along with the indicators *I cry and am moved easily*, *I feel more irritated than before*, *I feel sad* and *I feel scared*, showed emotional lability only contained by hypercontrol which remained with high values, but without differences, between the three groups/periods of time. Hence, there has been a shift from a very controlled way of coping with a possibility of escape through avoidance and certain flexibility through cognitive redefinition in the first group/period to a strategy of rigid control that considerably increases tension (*I do not sleep as well as before*, *My body is tense*). Ultimately, when it becomes unmanageable, it leads to lack of control in the third group/period.

The strategy *I speak to someone who can help me when the situation overwhelms me* showed high values in the three periods with non-significant differences. It indicated a search for help along with the belief that having a support group and psychological help would help them with their problems, which is probably not found among the groups of psychological support that were offered to them.

Moreover, due to the evidence that shows that women are a higher risk population than men (Lee et al., 2007; Lai et al., 2020), when they have to face this type of threats, the values of the indicators of depression, anxiety, intolerance of uncertainty, and coping strategies were compared in the two genders and in the three periods. Indeed, women obtained higher values than men in all the indicators of depression, except for *“I feel sad.”* For this indicator, although in the first period women obtained significantly higher values than men, this distance between the values of men and women was shortened until it disappeared in the third period, with men obtaining a slightly higher value than women.

Women also obtained higher values on the indicators of anxiety during the three periods, although it is important to note that the indicator *My body is tense* showed very high values in both genders. Women also obtained higher values than men with regard to the indicators of intolerance of uncertainty in all the periods, although the values were increased in both genders in the third period and in items such as: *Unexpected events bother me a lot*, and *I feel that even with the best planning, a small unexpected eventuality might ruin it all*, which also shows a lot of tension and irritability. Along the same line, Di Trani et al. (2021) observed that women scored higher in uncertainty avoidance and paralysis when facing it. These authors hypothesized that intolerance to uncertainty would serve as a moderator in the relationship between resilience and burnout. Finally, the coping profile of men and women has also shown significant differences, especially in *I burst out over anything*, in which women have significantly higher values than men. Despite this, both genders reached high values by the last period. Although both genders reached high values, men obtained significantly higher values than women with regard to the statement *I try to control my emotions*, especially in the third period.

Therefore, by taking gender into account, a coping profile that is similar to the one described for the general sample was found, with men being more hypercontrolled and women being more fragile due to the greater lack of affective control.

### Limitations

The present study has some limitations that must be taken into consideration. First, data obtained from self-reported questionnaires could facilitate social desirability rather than what their accurate response would be. Another limitation refers to the anonymity of the answers due to it being impossible to carry out a longitudinal study. Therefore, the type of design was cross-sectional (i.e., successive cross-sectional studies) and results should be interpreted with caution. Particularly, as associations and not as causality findings. Finally, due to the need for social distancing, the psychological evaluation was based on an online survey and self-reports. In future studies, it is recommended to add, if possible, other ways of complimentary evaluation.

## Concluding Remarks

In all cases, health personnel dedicated to the treatment of patients with COVID-19 shows higher rates of depression, anxiety, and intolerance of uncertainty. These values, according to the data collected at three different time periods discussed above, are shown to reach even higher, alarming limits. These differences in the psychological indicators have also led to differences in coping strategies, which continue to be dysfunctional. In fact, differences in strategies range from a way of coping with high control with mechanisms of avoidance and cognitive redefinition, to one which continues to have high mechanisms of control with rigid, excessive tension that seem to be more fragile due to the lack of flexibility (cognitive restructuring). Thereby, causing emotional outbursts when said coping strategies fail.

The importance of this study lies in the vital information it provides to know more about the mental health needs for the setting up of a large-scale therapeutic response during a sudden crisis. A rapid-response team in situations of crisis must include mental health workers. The medical staff, the nurses, and the personnel of local primary clinics in the epicenter of the crisis are fundamental for the general response (Kang et al., 2020). The effort in the psychological attention of these health personnel is essential to improve its immediate efficacy in the attention of infected patients and to better protect their mental health in the long haul.

The COVID-19 pandemic has revealed many problems regarding the supply of effective psychological interventions for health personnel. Governments should urgently establish active improvements in the intervention system based on solid scientific consultancy to effectively treat mental health problems of healthcare workers.

Finally, political decision-maker in charge of each section in a health agency should prioritize the psychological aspects of healthcare. Mental health should be a public health priority for both healthcare workers and the population in general.

## Data Availability Statement

The raw data supporting the conclusions of this article will be made available by the authors, without undue reservation.

## Ethics Statement

The project and questionnaire had the endorsement of the Research Ethics Committee of the Faculty of Health Sciences of the Adventist University of Plata, with No. CE000237 of the National Registry of Research in Health, and N° 3999 of Ministerial Resolution of the Ministry of Health of the Province of Entre Ríos, Argentina, Resolution 1.4/2020. The Informed consent was approved by the Research Ethics Committee, created by ministerial resolution 1002/16 and by the Personal Data Protection Law 25.326, which deals with the ethical implications of health research in which human beings participate, so as to protect their fundamental rights weighing, at the same time, the need to promote health research. The patients/participants provided their written informed consent to participate in this study.

## Author Contributions

MRi and RM: conceptualization, project administration, supervision, writing—original draft, and writing—review and editing. MK and MRo: data curation. MRi, BM, and VL: formal analysis. MK, JV, and MRo: investigation. MRi, RM, BM, VL, JV, and LE: methodology. MRi, RM, LE, JV, VL, BM, MK, and MRo: visualization. All authors contributed to the article and approved the submitted version.

## Conflict of Interest

The authors declare that the research was conducted in the absence of any commercial or financial relationships that could be construed as a potential conflict of interest.

## Publisher’s Note

All claims expressed in this article are solely those of the authors and do not necessarily represent those of their affiliated organizations, or those of the publisher, the editors and the reviewers. Any product that may be evaluated in this article, or claim that may be made by its manufacturer, is not guaranteed or endorsed by the publisher.
