# Supplementary material for: Perceived Concerns and Psychological Distress of Healthcare Workers Facing Three Early Stages of COVID-19 Pandemic
Source: Front Psychol. 2022 Mar 10;13:742810. doi: 10.3389/fpsyg.2022.742810 (PMC8965647; doi:10.3389/fpsyg.2022.742810)
Supplement: Supplementary file 1 [file Table_1.docx]

| Supplementary table | | | | | | |
| --- | --- | --- | --- | --- | --- | --- |
|  | | | | | | |
|  | N | Kkewness | | Kurtosis | |  |
|  | Statistical | Statistical | Standard error | Statistical | Estándar error |  |
| **Depression** |  |  |  |  |  |  |
| I am more irritated than before | 1458 | ,091 | ,064 | -,592 | ,128 |  |
| I feelsad | 1455 | -,111 | ,064 | -,582 | ,128 |  |
| I do not sleep as well as before | 1455 | -,084 | ,064 | -1,009 | ,128 |  |
| I feel guilty when I am resting | 1456 | ,722 | ,064 | -,559 | ,128 |  |
| **Anxiety** |  |  |  |  |  |  |
| I feelin secure | 1456 | ,094 | ,064 | -,802 | ,128 |  |
| I feels cared | 1456 | ,006 | ,064 | -,713 | ,128 |  |
| I feel discomfort in my stomach | 1455 | ,450 | ,064 | -,875 | ,128 |  |
| Mybodyis tense | 1455 | -,242 | ,064 | -,728 | ,128 |  |
| I cry or moved easily | 1456 | -,074 | ,064 | -1,009 | ,128 |  |
| I move and do things without and end in themselves | 1455 | ,429 | ,064 | -,777 | ,128 |  |
| **Intolerance of uncertainy** |  |  |  |  |  |  |
| I cannot be at peace if I do not know what will happen tomorrow | 1454 | ,137 | ,064 | -,984 | ,128 |  |
| Unexpected events bother me a lot | 1456 | -,090 | ,064 | -,798 | ,128 |  |
| I feel that even with the best planning, a small detail could ruin it all | 1457 | -,046 | ,064 | -,878 | ,128 |  |
| **Coping** |  |  |  |  |  |  |
| I focus exclusively in what I have to do, step by step | 1447 | -1,093 | ,064 | 1,222 | ,129 |  |
| I propose a different solution when the protocol fails | 1455 | -,599 | ,064 | ,152 | ,128 |  |
| I speak to someone who can help me when the situation overwhelms me | 1447 | -,516 | ,064 | -,363 | ,129 |  |
| I try to bring something positive out of the situation | 1444 | -,618 | ,064 | -,089 | ,129 |  |
| I try not to think about what is happening | 1456 | ,190 | ,064 | -,613 | ,128 |  |
| I accept it since there is nothing I can do about it | 1444 | -,065 | ,064 | -,786 | ,129 |  |
| I burst out over anything | 1444 | ,488 | ,064 | -,441 | ,129 |  |
| I try to control my emotions | 1445 | -,552 | ,064 | ,295 | ,129 |  |
